# Combination of s-methyl cysteine and protocatechuic acid provided greater lipid-lowering and anti-inflammatory effects in mice liver against chronic alcohol consumption

**DOI:** 10.22038/ijbms.2021.56705.12660

**Published:** 2021-08

**Authors:** Chun-Che Lin, Ya-Chen Yang, Chia-Yu Chen, Mei-Chin Yin

**Affiliations:** 1 Center for Digestive Medicine, China Medical University Hospital, China Medical University, Taichung, Taiwan; 2 Department of Food Nutrition and Health Biotechnology, Asia University, Taichung, Taiwan; 3 Department of Gastroenterology, Asia University Hospital, Taichung, Taiwan; 4 Department of Medical Research, China Medical University Hospital, China Medical University, Taichung, Taiwan

**Keywords:** Ethanol, Hepatic steatosis, Myeloperoxidase, Protocatechuic acid, S-methyl cysteine

## Abstract

**Objective(s):**

Protective effects of s-methyl cysteine (SMC) alone, protocatechuic acid (PCA) alone, and SMC plus PCA against chronic ethanol consumption induced hepatic steatosis and inflammation were investigated.

**Materials and Methods:**

Mice were divided into six groups: normal diet (ND) group, Lieber-DeCarli liquid diet without ethanol (LD diet) group, LD diet with ethanol (LED diet) group, SMC group (LED diet plus 0.25% SMC), PCA group (LED diet plus 0.25% PCA), and SMC+PCA group (LED diet plus 0.125% SMC + 0.125% PCA). After 8 weeks of supplementation, blood and liver were used for analysis.

**Results:**

Biochemical and histological data showed that SMC plus PCA led to a greater reduction in lipid droplets in the liver than SMC or PCA treatment alone. SMC plus PCA resulted in greater suppression in hepatic mRNA expression of peroxisome proliferator-activated receptor-gamma, sterol regulatory element-binding protein 1c, stearoyl-CoA desaturase-1, cyclooxygenase-2, and myeloperoxidase than SMC or PCA treatment alone. SMC plus PCA led to a greater decrease in hepatic reactive oxygen species and inflammatory cytokine levels than SMC or PCA treatment alone.

**Conclusion:**

These novel findings suggest that the combination of SMC and PCA was a potent remedy for alcoholic liver disorders.

## Introduction

Chronic and excessive ethanol intake leads to massive energy absorption, which subsequently promotes lipogenesis and hepatic steatosis, also called fatty liver (1, 2). The accumulation of lipids in blood or organs contributes to the occurrence and development of hypertension, atherosclerosis, and stroke (3, 4). It is reported that the major lipogenic genes include peroxisome proliferator-activated receptor (PPAR)-gamma, sterol regulatory element-binding protein 1c (SREBP1c), acetyl-CoA carboxylase 1 (ACC1), stearoyl-CoA desaturase-1 (SCD1), and 3-hydroxy-3-methyl-glutaryl-coenzyme A reductase (HMGCR) (5, 6). In addition, inflammatory stress due to activation of myeloperoxidase (MPO) and cyclooxygenase (COX)-2, as well as overproduction of tumor necrosis factor (TNF)-alpha, interleukin (IL)-1beta, IL-6, and prostaglandin E_2_ (PGE_2_) plays a crucial role in the progression of ethanol-induced hepatotoxicity (7, 8). These above events not only induce hepatic cell apoptosis but also cause immune dysfunctions (9, 10). Thus, any agent(s) with the effects to decrease lipid deposit and mitigate inflammatory response may potentially benefit hepatic protection against ethanol.

S-methyl cysteine (SMC) is a water-soluble cysteine derivative. It is a natural component compound present in edible *Allium* plants such as garlic and onion. Our previous study reported that dietary SMC intake via attenuating oxidative and inflammatory injury effectively protected mice liver against ethanol caused acute hepatoxicity (11). Our other study indicated that dietary SMC could decrease hepatic lipid content through mediating lipid metabolism-associated enzymes such as fatty acid synthase, malic enzyme, HMGCR, and SREBPs in high-fat diet treated mice (12). Protocatechuic acid (PCA), a phenolic acid, naturally occurs in many edible plant foods such as *Hibiscus sabdariffa* and green tea (13, 14). A study (15) found that PCA could regulate both activity and mRNA expression of some lipogenic enzymes, and improve trans fatty acid-induced hepatic steatosis. Another study (16) revealed that oral administration of PCA lowered plasma and hepatic levels of triglyceride (TG) and total cholesterol (TC) in mice treated by D-galactosamine. Farombi *et al*. (17) reported that dietary PCA attenuated dextran sulfate sodium-induced hepatotoxicity through decreasing plasma levels of pro-inflammatory cytokines. Those previous studies suggest that SMC and PCA are potent natural protective agents for the liver. However, the combined effects of SMC plus PCA against alcoholic hepatic injury remain unknown. Several studies have indicated that the combination of a hydrophilic agent plus a lipophilic agent offered greater effects against diseases. For example, carnosine plus vitamin E exhibited greater protection for the heart against doxorubicin (18); SMC plus syringic acid displayed greater anti-seizure activities (19); PCA plus 5-fluorouracil caused greater apoptotic action in gastric adenocarcinoma cells (20). Obviously, the combination of two agents with different chemical characteristics may have more biochemical interactions, which in turn efficiently prevents or ameliorates diseases. 

In our current study, the hepatic protection of SMC alone, PCA alone, and SMC plus PCA against chronic ethanol consumption-induced steatosis and inflammation were investigated. The effects of these treatments upon lipid levels, oxidative and inflammatory indicators, as well as mRNA expression of associated genes, were examined. These novel findings could support the possibility of using SMC plus PCA to attenuate alcoholic hepatic injury.

## Materials and Methods

***Materials***

SMC (99%) and PCA (97%) were purchased from Wako Pure Chemical Co. (Tokyo, Japan). Lieber-DeCarli liquid diets were bought from Bioserve Inc. (Frenchtown, NJ, USA). C57BL/6 male mice at 4-wk old were obtained from National Laboratory Animal Center (Taipei City, Taiwan). The use of mice was reviewed and approved by the Asia University animal care and use committee(permission number, 106-asia-15). The regulations for laboratory animal’s use and care were strictly adopted. 

***Experimental design***

Mice were acclimated for one week and randomly divided into six groups: normal diet (ND) group, Lieber-DeCarli liquid diet without ethanol (LD diet) group, Lieber-DeCarli liquid diet with ethanol (LED diet) group, SMC group (LED diet plus 0.25% SMC), PCA group (LED diet plus 0.25% PCA), SMC+PCA group (LED diet plus 0.125% SMC + 0.125% PCA). Mice in the ND group consumed a standard basal diet. A liquid diet with or without ethanol was supplied in drinking bottles. SMC or PCA was mixed in the feed. In the LD diet, carbohydrates offered 47% of total energy. In the LED diet, ethanol and carbohydrates offered 36% and 11% of total energy, respectively. Mice body weight and consumed food intake were recorded. After 8 weeks of supplementation, all mice were overnight fasted, followed by carbon dioxide treatment for sacrification. Blood and liver were collected. Blood was centrifuged immediately to obtain plasma. The liver sample at 20 mg was dissected and mixed with 2.5 ml phosphate-buffered saline (PBS, pH 7.2), and followed by homogenization. The protein concentration of plasma and liver homogenate was determined by an assay kit (Cat. No. 23225, Pierce, Rockford, IL, USA). Bovine serum albumin was used as a standard for protein concentration determination.

***Blood analysis***

Plasma alanine transaminase (ALT) and aspartate transaminase (AST) activities were measured by assay kits purchased from Randox Lab. Ltd. (Cat. No. AL1268 and AS1267, Crumlin, UK). Plasma levels of TG and TC were quantified by assay kits obtained from Sigma Chem. Co. (Cat. No. MAK264 and MAK043, St. Louis, MO, USA). 

***Hepatic lipid analysis***

Liver homogenate at 1 ml was mixed with a solution containing chloroform and methanol (2:1, v:v). The chloroform layer was collected after a 3-min shaking, and followed by using a rotary evaporator (Evela, Rikakikai Co., Tokyo, Japan) to concentrate. Then, the concentrate was mixed with a Triton X-100 isopropanol solution. TG and TC content was assayed by E-test kits purchased from Wako Pure Chemical Co. (Cat. No. 290-63701 and 439-17501, Tokyo, Japan). 

***Histological assay for lipid accumulation***

The histological assay was processed according to the method of Ren *et al*. (21). Hepatic tissue was excised and washed twice with ice-cold PBS. Then, the liver sample was immediately frozen by liquid nitrogen. After 30 min liquid nitrogen treatment, the frozen liver sample was cut to 6-μm thick sections. Sections were fixed in 10% formalin for 5 min at 4 °C followed by incubating with freshly prepared Oil Red O stain solution (Sigma-Aldrich, St. Louis, MO, US) at room temperature in the dark for 10 min. After washing twice with deionized water, the sample was counterstained with hematoxylin at room temperature for 5 min. These sections were observed under a light microscope (model Olympus CKX51, Olympus Co. Tokyo, Japan). More red dots represented more lipid droplets. 

***Assays of oxidative and inflammatory mediators***

Hepatic reactive oxygen species (ROS) level was determined by treating liver homogenate with 2’,7’-dichlorofluorescein diacetate. After 30 min incubation at 37 °C, the change in fluorescence value at both 525 nm and 488 nm was monitored by a microplate reader (Molecular Devices, Sunnyvale, CA, USA). Data were expressed as relative fluorescence unit (RFU)/mg protein. Hepatic content of glutathione (GSH) and PGE_2_ was determined by kits purchased from Cayman Chem. Co. (Cat. No. 703002 and 514010, Ann Arbor, MI, USA). Hepatic levels of TNF-alpha, IL-1beta, and IL-6 were quantified by assay kits obtained from BioLegend (Cat. No. 430904, 432604 and 431307, San Diego, CA, USA). Hepatic COX-2 activity (U/mg protein) was measured by a commercial kit (Cat. No. 700200, Cayman Chem. Co., Ann Arbor, MI, USA). Hepatic MPO activity was detected according to the method of da Silva-Santi *et al*. (22). In brief, the hepatic homogenate was centrifuged 500 xg at 4 °C for 5 min. Then, supernatant at 10 µl was mixed with 200 µl PBS containing o-dianisidine dihydrochloride and 1% H_2_O_2_. Sodium acetate was added to stop the enzyme reaction, and MPO activity was assayed by monitoring the absorbance at 460 nm. Data were expressed as folds of the ND group.

***Measurement of hepatic mRNA expression***

mRNA expression of target genes was quantified by real-time polymerase chain reaction (RT-PCR). TRIzol^®^ reagents (Life Technologies, Grand Island, NY, USA) were used to extract hepatic total RNA, and the concentration of total RNA was determined by measuring the absorbance at 260 nm. RNA at 2 µg was further used to synthesize cDNA by a commercial cDNA synthesis kit (Cat. No. 629020, Legene Biosciences, San Diego, CA, USA). Then, the synthesized cDNA was further used for performing RT-PCR. RT-PCR was handled by an AB7500 real-time PCR system (Applied Biosystems, Foster City, CA, USA), in which SYBR Premix Ex TaqTM II was used as the fluorescent dye. The used PCR primers for target genes are shown in Table 1. The mRNA expression was normalized against a housekeeping gene, glyceraldehyde-3-phosphate dehydrogenase (GAPDH).

***Statistical analysis***

Data, expressed as means ± standard deviation (SD), were obtained from eight mice (n=8). Those data were subjected to one-way ANOVA analysis. Tukey’s post-test followed to evaluate the differences among groups. *P*-value<0.05 was defined as statistically significant. 

## Results

***SMC and PCA improved liver weight and functions***

As shown in Table 2, ethanol intake decreased body weight gain, increased liver weight, and raised plasma ALT and AST activities (*P*<0.05). SMC or PCA supplement alone reversed these changes (*P*<0.05). SMC plus PCA led to lower liver weight and lower plasma ALT and AST activities than SMC or PCA treatment alone (*P*<0.05).

***SMC and PCA decreased hepatic lipid accumulation***

**Figure 1.**
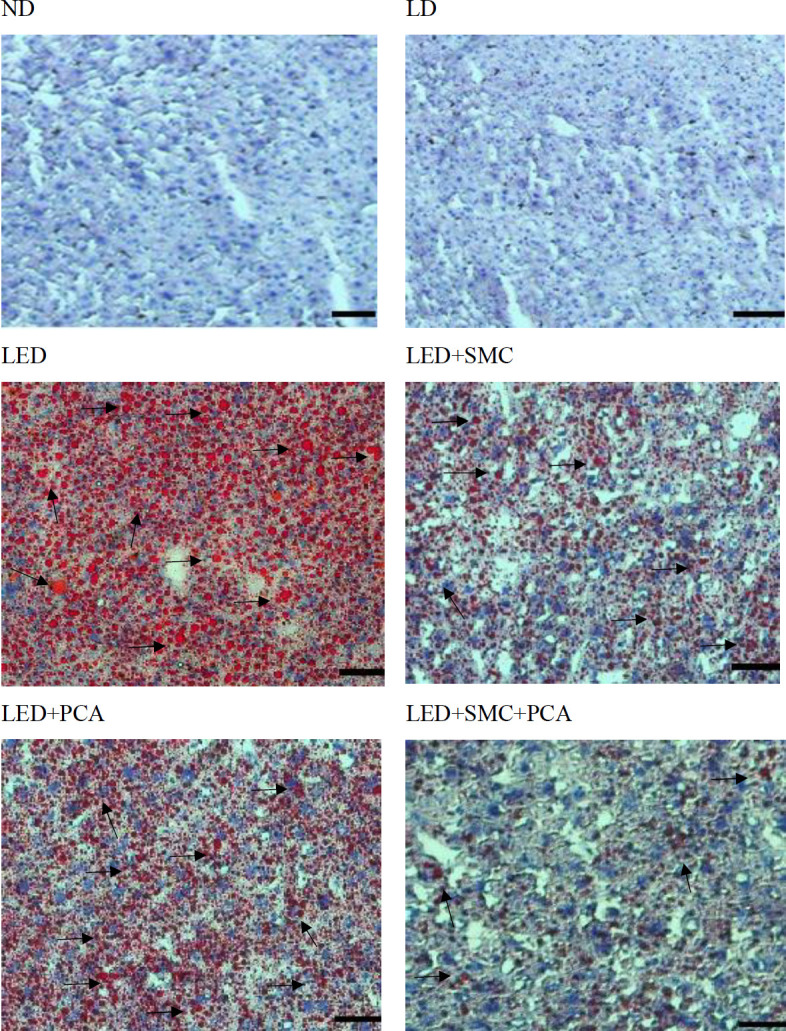
Effects of s-methyl cysteine (SMC) and/or Protocatechuic acid (PCA) upon hepatic lipid accumulation, determined by Oil Red O (ORO) statin, in mice treated with normal diet (ND), liquid diet (LD, Lieber–DeCarli liquid diet without ethanol), ethanol diet (LED, Lieber–DeCarli liquid diet with ethanol), LED+SMC (0.25%), LED+PCA (0.25%) and LED+SMC (0.125%)+PCA (0.125%) for 8 weeks. Magnification: 200X (scale bar = 50 µm). Red oil droplets are indicated by arrow bars

Ethanol intake increased TG and TC content in plasma and liver (Table 3, *P*<0.05). SMC or PCA treatment alone decreased TG and TC content in plasma and liver (*P*<0.05). SMC plus PCA resulted in more reduction in plasma and hepatic TG levels than SMC or PCA treatment alone (*P*<0.05). Histological results are shown in Figure 1, and arrow bars are used to indicate red oil droplets. Ethanol intake caused excessive hepatic lipid accumulation because of the presence of more and big red oil droplets when compared with a liver sample from the ND group. SMC or PCA treatment alone diminished hepatic lipid deposits and resulted in fewer red oil droplets when compared with LED groups. The combination of SMC plus PCA led to lower red oil droplets than SMC or PCA treatment alone.

***SMC and PCA mediated hepatic mRNA expression***

**Figure 2:**
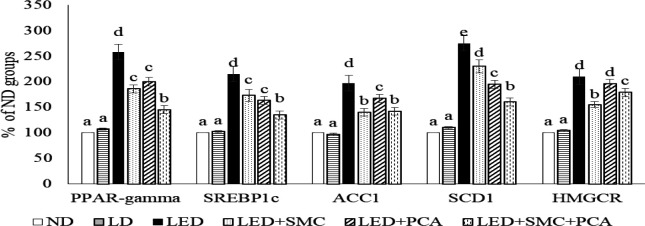
Hepatic mRNA expression of lipogenic genes, peroxisome proliferator-activated receptor (PPAR)-gamma, sterol regulatory element-binding protein (SREBP)1c, acetyl-CoA carboxylase (ACC)1, stearoyl-CoA desaturase (SCD)1 and 3-hydroxy-3-methyl-glutaryl-coenzyme A reductase (HMGCR), in mice treated with normal diet (ND), liquid diet (LD, Lieber–DeCarli liquid diet without ethanol), ethanol diet (LED, Lieber–DeCarli liquid diet with ethanol), LED+ s-methyl cysteine (SMC) (0.25%), LED+ Protocatechuic acid (PCA) (0.25%) and LED+SMC (0.125%)+PCA (0.125%) for 8 weeks. Values are mean±SD, n=8. a-eValues among bars without a common letter differ, *P*<0.05

Ethanol enhanced hepatic mRNA expression of PPAR-gamma, SREBP1c, ACC1, SCD1, and HMGCR (Figure 2, *P*<0.05). SMC treatment alone suppressed mRNA expression of these five factors (*P*<0.05). Besides HMGCR, PCA treatment alone limited mRNA expression of the other four factors (*P*<0.05). SMC plus PCA led to a greater decrease in mRNA expression of PPAR-gamma, SREBP1c, and SCD1 than SMC or PCA treatment alone (*P*<0.05). 

***SMC and PCA mitigated hepatic oxidative and inflammatory stresses***

**Figure 3:**
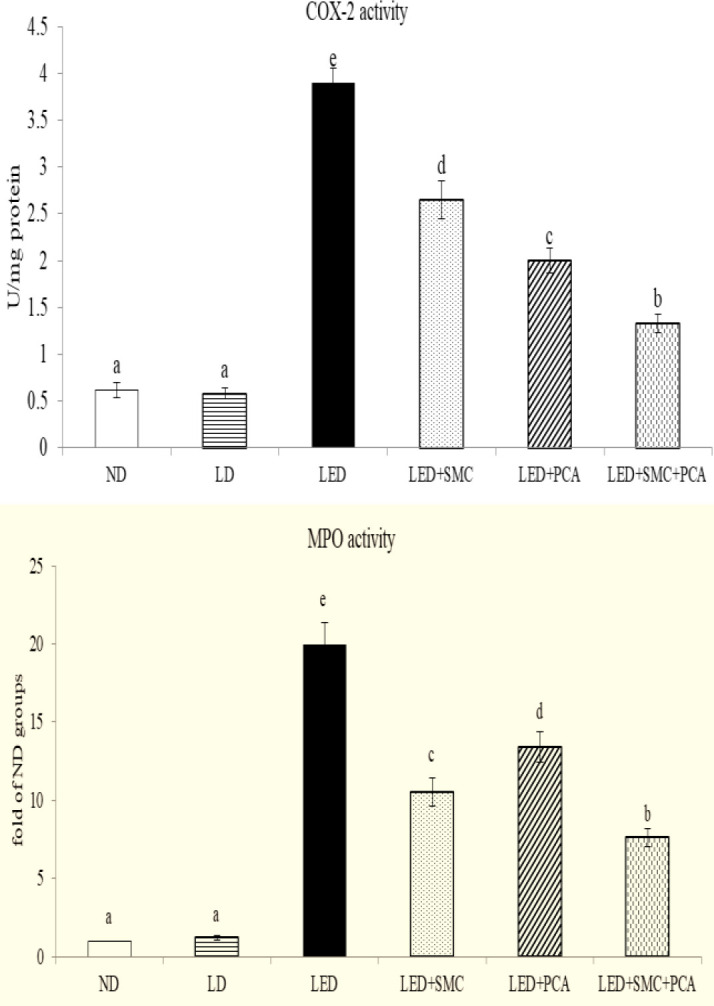
Hepatic activity (fold of ND groups) of cyclooxygenase (COX)-2 (U/mg protein) and myeloperoxidase (MPO) in mice treated with normal diet (ND), liquid diet (LD, Lieber–DeCarli liquid diet without ethanol), ethanol diet (LED, Lieber–DeCarli liquid diet with ethanol), LED+ s-methyl cysteine (SMC) (0.25%), LED+PCA (0.25%) and LED+SMC (0.125%)+PCA (0.125%) for 8 weeks. Values are mean±SD, n=8. a-e Values among bars without a common letter differ, *P*<0.05

**Figure 4:**
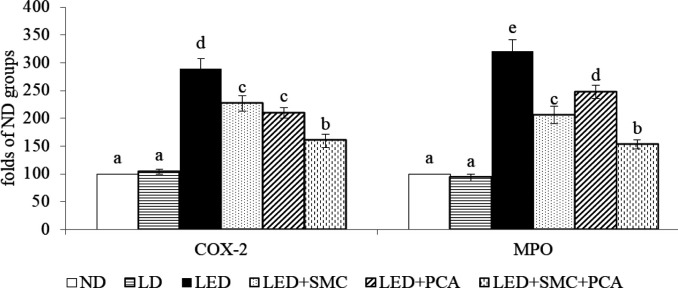
Hepatic mRNA expression of cyclooxygenase (COX)-2 and myeloperoxidase (MPO) in mice treated with normal diet (ND), liquid diet (LD, Lieber–DeCarli liquid diet without ethanol), ethanol diet (LED, Lieber–DeCarli liquid diet with ethanol), LED+SMC (0.25%), LED+ Protocatechuic acid (PCA) (0.25%) and LED+ s-methyl cysteine (SMC) (0.125%)+PCA (0.125%) for 8 weeks. Values are mean±SD, n=8. a-e Values among bars without a common letter differ, *P*<0.05

Ethanol intake increased hepatic ROS level and decreased hepatic GSH content (Table 4, *P*<0.05). SMC or PCA treatments reversed these changes (*P*<0.05). SMC plus PCA led to a greater reduction in ROS level than SMC or PCA treatment alone (*P*<0.05). Ethanol intake increased hepatic release of TNF-alpha, IL-1beta, IL-6, and PGE_2_ (*P*<0.05). SMC or PCA treatment alone lowered the levels of these inflammatory factors, in which PCA was greater than SMC in limiting PGE_2_ generation (*P*<0.05). SMC plus PCA led to a greater decrease in these four inflammatory factors (*P*<0.05). As shown in Figure 3, ethanol enhanced hepatic COX-2 and MPO activities (*P*<0.05). SMC or PCA treatment alone diminished both COX-2 and MPO activities in the liver (*P*<0.05), in which PCA was better than SMC in reducing COX-2 activity; and SMC was better than PCA in declining MPO activity (*P*<0.05). SMC plus PCA resulted in lower COX-2 and MPO activities than SMC or PCA treatment alone (*P*<0.05). Ethanol up-regulated hepatic mRNA expression of COX-2 and MPO (Figure 4, *P*<0.05). SMC or PCA treatment alone suppressed mRNA expression of these two factors, in which SMC was greater than PCA in down-regulating MPO mRNA expression (*P*<0.05). SMC plus PCA led to greater suppression in hepatic mRNA expression of COX-2 and MPO than SMC or PCA treatment alone (*P*<0.05).

## Discussion

Alcoholic fatty liver disease is a major cause of hepatitis, cirrhosis, and even hepatocellular carcinoma. It is also highly associated with non-hepatic complications including hypertension, diabetes, kidney disease, and cardiovascular disorders (23). Thus, any agent(s) with the potential to alleviate alcoholic fatty liver disease may also protect other organs or systems. The hepatic anti-inflammatory protection from SMC or PCA has been reported (11, 17). Thus, it was not surprising to observe lower ALT and AST activities in mice with SMC or PCA treatments. Our present study did not intend to compare the protective difference between these two agents. This study focused on the combined effects of these two agents for the liver against ethanol-induced injury. Our data revealed that this combination provided greater effects in lowering lipid accumulation, attenuating inflammatory stress, as well as mediating both activity and mRNA expression of associated factors for the liver against ethanol. These findings suggested that SMC plus PCA was a more effective remedy against alcoholic hepatic disorders. 

It is reported that cysteine derivative was a strong nucleophile, and addition of cysteine derivative to PCA resulted in higher radical scavenging equivalence than PCA alone (24). Thus, it was possible that SMC, a cysteine derivate, enhanced the anti-oxidative efficiency of PCA, which consequently offered greater protective activities as we observed in the combination of SMC plus PCA. In addition, it is speculated that a hydrophilic agent like SMC exerts its bioactivities in nucleoli and aqueous fractions; and a lipophilic agent like PCA exerts its bioactivities in lipidic fractions. Consequently, this combination provided more efficient protection for both aqueous and lipidic fractions of hepatocytes. 

As reported by others (1, 25), chronic ethanol consumption increased lipid deposits in circulation and the liver. Ethanol exposure impaired hepatic lipid homeostasis via promoting SREBP-1c-driven *de novo* lipogenesis and limiting PPAR-alpha mediating mitochondrial beta-oxidation and lipolysis, which finally resulted in the accumulation of lipid molecules, mainly TG, in hepatocytes (26, 27). Our histological data agreed with those previous studies and revealed that ethanol intake led to massive red oil droplets in LED groups. Furthermore, our results indicated that SMC or PCA treatments limited hepatic mRNA expression of four crucial lipogenic genes including PPAR-gamma, SREBP1c, ACC1, and SCD1, which in turn reduced hepatic TG biosynthesis. In addition, we found that SMC plus PCA caused greater suppressive effects upon mRNA expression of PPAR-gamma, SREBP1c, and SCD1, which definitely contributed to restricting lipogenesis and decrease TG generation in the liver. Our histological data also agreed that this combination, SMC plus PCA, markedly reduced hepatic red lipid droplets. These data support and explain that this combination was more effective in alleviating ethanol-induced hepatic steatosis. Since hepatic steatosis in this SMC plus PCA group has been improved, the observed lower liver weight could be explained. A study (28) reported that the periportal and perivenous zones of hepatocytes display an obvious heterogeneity in gene expression. Another study (29) indicated that cholesterol metabolism-related genes were expressed in periportal hepatocytes, and other lipogenic genes were mostly expressed in the perivenous zone. In our present study, SMC, not PCA, could mediate mRNA expression of HMGCR, an important enzyme involved in cholesterol biosynthesis. It seems that SMC, not PCA, could access the periportal zone. Thus, the affinity of SMC plus PCA to the periportal zone might be relatively weaker than SMC treatment alone, which consequently declined the regulatory ability of this combination upon mRNA expression of HMGCR. On the other hand, we found that SMC alone, PCA alone, and SMC plus PCA could mediate other lipogenic genes. It is highly possible that both compounds could access the perivenous zone of hepatocytes and mediate the genes responsible for TG biosynthesis. 

Ethanol enhanced homocysteine accumulation and cysteine shortage, which in turn resulted in GSH depletion and increased oxidative stress (30, 31). It is reported that SMC or PCA provided anti-oxidative activities such as increasing GSH levels in rodents (32, 33). Thus, it seems not surprising to observe the higher GSH level and lower ROS level in the liver of SMC or PCA-treated mice. However, our results indicated that SMC plus PCA led to a more efficient increase in hepatic GSH level, which consequently attenuated ethanol-induced hepatic oxidative stress. The observed diminished oxidative stress not only benefited hepatic functions such as lipid metabolism but also contributed to decrease the release of inflammatory cytokines in the liver (34). As reported by others (35, 36), ethanol intake caused hepatic inflammatory injury. Our data revealed that ethanol intake promoted the activity and mRNA expression of COX-2 and MPO, two inflammatory indicators, which subsequently facilitated the production of TNF-alpha, IL-1beta, IL-6, and PGE_2_ in the liver. It is well known that these mediators are responsible for inflammatory progression (37). However, we found that SMC plus PCA substantially limited both activity and mRNA expression of COX-2 and MPO, which consequently reduced the production of inflammatory cytokines. MPO is an inflammatory enzyme mainly formed by polymorphonuclear neutrophils (38). Increased activity and expression of MPO stimulate the release of pro-inflammatory mediators and cytotoxic oxidants (39). In our present study, SMC plus PCA markedly suppressed both activity and mRNA expression of MPO in the liver, which in turn diminished the generation of cytokines and oxidants. These results suggest that this combination could more effectively improve ethanol-induced hepatic oxidative and inflammatory damage.

 It has been attractive to use phytochemicals such as crocin or thymoquinone to prevent or alleviate ethanol-induced liver damage (40, 41). Both SMC and PCA are natural compounds present in some plant foods, and their pharmacological potentials against insulin resistance and hypertension have been paid attention to (32, 33). Based on their natural properties, the consumption of these compounds might be safe. The used dosage in this combination for mice was equal to 7 g SMC plus 7 g PCA per day for an adult man with a 70-kg body weight. These dosages seem feasible for humans. However, further studies are necessary to ensure the efficiency and safety of this combination. Besides greater protection, combined use of two or more natural compounds might have other advantages such as lower cost and fewer side effects when compared with one single agent at high doses. 

**Table 1:** Primer sequences of target genes for RT-PCR analysis ???

Target		Primer
PPAR-gamma	F	5′-GAG TGT GAC GAC AAG ATT TG-3′
	R	5′-GGT GGG CCA GAA TGG CAT CT-3′
SREBP1c	F	5′-GGA GCC ATG GAT TGC ACA TT-3′
	R	5′-CCT GTC TCA CCC CCA GCA TA-3′
ACC1	F	5′-GGA CAG ACT GAT CGC AGA GAA AG-3′
	R	5′-TGG AGA GCC CCA CAC ACA-3′
SCD1	F	5′-CCC CTG CGG ATC TTC CTT AT-3′
	R	5′-AGG GTC GGC GTG TGT TTC T-3′
HMGCR	F	5′-TTC ACG CTC ATA GTC GCT GGA TAG-3′
	R	5′-TGG TTC AAT TCT CTT GGA CAC ATC TTC-3′
COX-2	F	5’-GCC TGG TCT GAT GAT GTA TGC-3’
	R	5’-GAG TAT GAG TCT GCT GGT TTG G-3’
MPO	F	5’-GGA AGG AGA CCT AGA GGT TGG-3’
	R	5’-TAG CAC AGG AAG GCC AAT G-3’
GAPDH	F	5’-GGA TGC AGG GAT GAT GTT C-3’
	R	5’-TGC ACC ACC AAC TGC TTA G-3’

PPAR: peroxisome proliferator-activated receptor; SREBP: sterol regulatory element-binding protein; ACC: acetyl-CoA carboxylase; SCD: stearoyl-CoA desaturase; HMGCR: 3-hydroxy-3-methyl-glutaryl-coenzyme A reductase; COX: cyclooxygenase; MPO: myeloperoxidase; GAPDH: glyceraldehyde-3-phosphate dehydrogenase

**Table 2:** Initial body weight (IBW, g/mouse), final body weight (FBW, g/mouse), BW gain (g/mouse), food intake (FI, g/mouse/day), liver weight (LW, g), and plasma activity (U/L) of alanine transaminase (ALT) and aspartate transaminase (AST) in mice treated with normal diet (ND), liquid diet (LD, Lieber–DeCarli liquid diet without ethanol), ethanol diet (LED, Lieber–DeCarli liquid diet with ethanol), LED+SMC (0.25%), LED+PCA (0.25%) and LED+SMC (0.125%)+PCA (0.125%) for 8 weeks

	ND	LD	LED	LED+SMC	LED+PCA	LED+SMC+PCA
IBW	20.9 1.0^a^	19.8 1.2^a^	20.6 0.8^a^	20.2 1.3^a^	19.7 0.9^a^	21.0 1.1^a^
FBW	28.1 1.4^c^	30.5 1.8^d^	24.4 1.0^a^	26.7 0.9^b^	26.4 0.5^b^	27.7 1.2^c^
BW gain	6.9 0.5^c^	8.3 0.4^d^	3.8 0.3^a^	5.4 0.6^b^	5.8 0.5^b^	6.0 0.4^b^
FI	2.2 0.6^a^	2.4 0.7^a^	2.1 0.3^a^	2.2 0.4^a^	2.1 0.5^a^	2.3 0.4^a^
LW	1.41 0.09^a^	1.48 0.1^a^	1.82 0.13^c^	1.65 0.07^b^	1.69 0.05^b^	1.53 0.08^a^
ALT	21 4^a^	23 2^a^	95 8^d^	54 6^c^	60 4^c^	45 5^b^
AST	19 3^a^	22 4^a^	109 10^d^	67 3^c^	62 6^c^	42 4^b^

Values are mean ± SD, n=8. a-dValues in a row without a common letter differ, *P*<0.05

**Table 3:** Plasma level (g/l) of triglyceride (TG) and total cholesterol (TC), and hepatic level (mg/g wet weight) of TG and TC in mice treated with normal diet (ND), liquid diet (LD, Lieber–DeCarli liquid diet without ethanol), ethanol diet (LED, Lieber–DeCarli liquid diet with ethanol), LED+SMC (0.25%), LED+PCA (0.25%) and LED+SMC (0.125%)+PCA (0.125%) for 8 weeks

	ND	LD	LED	LED+SMC	LED+PCA	LED+SMC+PCA
Plasma						
TG	2.27 0.14^a^	2.34 0.17^a^	4.85 0.23^e^	3.59 0.15^c^	4.04 0.09^d^	3.06 0.11^b^
TC	1.09 0.05^a^	1.18 0.1^a^	2.19 0.08^d^	1.73 0.05^b^	1.98 0.07^c^	1.91 0.9^c^
Hepatic						
TG	21.7 1.6^a^	24.0 1.2^a^	45.8 2.1^e^	34.7 1.5^c^	38.0 1.8^d^	30.2 1.3^b^
TC	2.79 0.08^a^	2.83 0.13^a^	4.21 0.19^d^	3.34 0.15^b^	3.87 0.1^c^	3.71 0.12^c^

Values are mean ±SD, n=8. a-e Values in a row without a common letter differ, *P*<0.05

**Table 4:** Hepatic level of reactive oxygen species (ROS), glutathione (GSH), tumor necrosis factor (TNF)-alpha, interleukin (IL)-1beta, IL-6 and prostaglandin E (PGE)2 in mice treated with normal diet (ND), liquid diet (LD, Lieber–DeCarli liquid diet without ethanol), ethanol diet (LED, Lieber–DeCarli liquid diet with ethanol), LED+SMC (0.25%), LED+ Protocatechuic acid (PCA) (0.25%) and LED+SMC (0.125%)+PCA (0.125%) for 8 weeks

	ND	LD	LED	LED+SMC	LED+PCA	LED+SMC+PCA
ROS, RFU/mg protein	0.27 0.04^a^	0.22 0.05^a^	1.78 0.15^d^	1.19 0.12^c^	1.28 0.08^c^	0.89 0.1^b^
GSH, nmol/mg protein	13.1 0.8^d^	12.9 0.6^d^	6.9 0.4^a^	9.7 0.7^c^	8.1 0.5^b^	10.8 0.6^c^
TNF-alpha, pg/mg protein	22 4^a^	25 3^a^	191 18^d^	122 10^c^	139 14^c^	97 8^b^
IL-1beta, pg/mg protein	19 3^a^	23 5^a^	150 13^d^	103 9^c^	97 12^c^	75 5^b^
IL-6, pg/mg protein	18 4^a^	21 2^a^	162 17^d^	110 14^c^	104 8^c^	83 6^b^
PGE_2_, pg/g protein	588 36^a^	602 41^a^	1937 115^e^	1493 80^d^	1172 94^c^	951 58^b^

Values are mean±SD, n=8. a-e Values in a row without a common letter differ, *P*<0.05

## Conclusion

The combination of SMC and protocatechuic acid provided greater effects to improve hepatic steatosis and inflammatory stress in mice with chronic ethanol consumption than SMC or protocatechuic acid treatment alone. This combination effectively mediated hepatic mRNA expression of associated factors. These findings suggest that this combination was a novel and potent remedy for alcoholic liver disorders.
